# The usefulness of a free self-test for screening albuminuria in the general population: a cross-sectional survey

**DOI:** 10.1186/1471-2458-9-381

**Published:** 2009-10-09

**Authors:** Markus MJ Nielen, François G Schellevis, Robert A Verheij

**Affiliations:** 1 NIVEL (Netherlands Institute for Health Services Research), P.O. Box 1568, 3500BN Utrecht, The Netherlands; 2 Department of General Practice/EMGO Institute, VU University Medical Center, Amsterdam, The Netherlands

## Abstract

**Background:**

In this study we evaluated the usefulness of a free self-test for screening albuminuria in the general population.

**Methods:**

Dutch adults were invited by the Dutch Kidney Foundation to order a free albuminuria self-test, consisting of three semi quantitative dipstick tests, via the Internet. Results were classified in negative, low-positive and high-positive. In case of a positive test result, the tester was recommended to visit a GP for supplementary examination and/or treatment. Participants of the programme were sent a questionnaire for evaluation by e-mail eight weeks after receiving the self-test.

**Results:**

During the first 30 days of the self-test programme, 996,927 self-tests were ordered. In total, 71,714 participants completed the questionnaire: 79% had a negative test result and 21% had a positive test result (20% low-positive and 1% high-positive). Of the positive testers, 25% visited a GP after testing for albuminuria. Among the 3,983 participants who visited a GP, 193 new diseases were detected: 25 chronic renal failure, 152 hypertension and 31 diabetes mellitus.

**Conclusion:**

Using a free self-test for screening albuminuria in the general population resulted in a large response and a number of newly detected diseases. However, we found a very high percentage of positive testers of which probably a large part is false positive. Furthermore, only a small part of the positive testers visited a GP for additional examination and/or treatment. The efficiency of such a campaign could be increased by embedding the testing in health care to reduce the number of false-positive results and to ensure follow-up and treatment in case of a positive test result.

## Background

The prevalence rate of chronic diseases like cardiovascular diseases and diabetes mellitus is rapidly increasing, particularly in industrialized countries [1,2]. The perceived need for early detection of chronic diseases is growing to prevent future problems for the patient and to reduce the costs for health care. Therefore, screening for chronic disorders in the general population becomes more popular.

In 2006, the Dutch Kidney Foundation started a program to detect persons at risk for chronic renal failure (CRF) in the Netherlands by offering a free self-test for albuminuria via the Internet. CRF is the irreversible loss of kidney function and can result in total loss of kidney function, necessitating dialysis or kidney transplantation to prevent death [3]. Dialysis and transplantation not only have a big impact on patients, but also lead to high costs. In the Netherlands, the annual health care costs of end-stage CRF patients amount to more than 400 million euros [4]. CRF also increases the risk for cardiovascular morbidity and mortality [5].

The early stages of CRF are asymptomatic. Since symptoms of CRF, like fatigue and weakness, only start when the kidney function is decreased by about 60-70%, it is difficult to detect the disease at an early stage [3]. The presence of an increased amount of protein in the urine (albuminuria) is an indicator for worsening kidney function and increases the risk of developing CRF. Patients with macroalbuminuria (albuminuria concentration > 200 mg/l) have a three times higher risk for dialysis or kidney transplantation within 5 to 15 years [6]. When macroalbuminuria is detected at an early stage of CRF, treatment with an angiotensin-receptor blocker (ARB) or angiotensin converting enzyme (ACE) inhibitor can prevent or delay the development of end stage kidney disease [7,8]. Due to the high impact of end-stage CRF for patients and health care, the symptom-less early stages of the disease and the availability of preventive treatment, mass screening for CRF can be justified according to the criteria of Wilson and Jungner [9].

In the program, an albuminuria self-test was used as screening tool. The popularity of self-tests is increasing. Ryan et al. identified 104 self tests for 24 diseases, including cancers, chronic diseases and infection, available on the Internet according to a systematic Internet search [10]. People are increasingly interested in their health and with a self-test an individual can obtain information without the help of a health professional. Besides the benefit of privacy, there are also disadvantages of the increasing popularity of self-tests. The most important disadvantage of self-tests is the number of false-positive test results and its consequences in terms of unnecessary diagnostics, unnecessary costs, medicalization and patient worries.

In the present study we evaluate the usefulness of a free self-test for screening of albuminuria in the general population. We investigated the following research questions: 1) what are the characteristics of the persons who ordered a self-test for albuminuria?, 2) how many persons had a positive test result on the albuminuria self-test?, 3) how many persons visited a GP after testing positive for albuminuria?, and 4) how many persons with CRF and risk factors for CRF (hypertension and diabetes mellitus) were detected by the GP via the albuminuria self-test program?

## Methods

### Albuminuria self-test program

The Dutch Kidney Foundation started a screening program for CRF in September 2006. With advertisements on the radio, television, newspapers and the Internet, all Dutch adults were invited to test for albuminuria with a free self-test, called the 'Kidney check' (in Dutch 'Niercheck'). The self-test could be ordered via the website of the Dutch Kidney Foundation after registration of name, address, age, gender and e-mail address, or by telephone. Since a minority of the self-tests was ordered by telephone, only data from the participants who ordered the self-test via the Internet were used. The Internet order form included a question about the willingness to complete a questionnaire for evaluation of the self-test program (informed consent). Notification for this questionnaire was by e-mail. According to Dutch legislation, approval by a medical ethics committee was not needed.

### Self-test for albuminuria

The albuminuria self-test consisted of three semi quantitative dipstick tests (Machery-Nagel: Düren, Germany) accompanied with a manual and a colour chart to determine the test result. On three different days, with at least five days in between, the first voided morning urine sample should be used for testing. In the manual it was advised not to test during pregnancy or menstruation, after heavy exercise or when suffering from influenza-like-illness. After applying the dipstick test, the colour of the stick indicated the level of albumin in the urine: 0, 30, 100 or 500 mg/l albumin respectively. To determine the test result reliably, it was advised to read the test result in daylight. Since a single dipstick test has a sensitivity of 96% and a specificity of 85%, the total test result was considered positive (i.e. elevated albumin levels in the urine) when in at least two out of three dipstick tests a concentration of 30 mg/l or higher was found. In case of a positive test result, the tester was recommended to visit his/her GP for additional examination and/or treatment. We classified the test results as negative, low-positive (at least two positive dipstick tests; highest test result 30 mg/l) and high-positive (at least two positive test results; at least one test result of 100 mg/l or 500 mg/l).

### Study population

All participants who gave permission, were sent a link for a web-questionnaire by e-mail (personal URL for on-line questionnaire) eight weeks after the self-test had been sent. The questionnaire included questions about: 1) demographic characteristics: age, gender, level of education and postal code, 2) medical history: self-reported presence of cardiovascular diseases, diabetes mellitus, kidney disease, alcohol use, smoking history and body mass index (BMI), 3) the results of the dipstick tests, and 4) information about GP consultations: detection of hypertension, diabetes mellitus and/or a kidney disease.

### Statistical analyses

Frequencies were calculated for the number of persons with a negative, low-positive and high-positive test result in the total group of participants, by age group (18-29 years, 30-39 years, 40-49 years, 50-59 years, 60-69 years and 70 years and older) and for persons with or without risk factors at baseline (diabetes mellitus and/or hypertension), respectively. Next, the number of patients who visited a GP after performing the self-test was calculated per subgroup of the test result. Also, the number of detected persons with CRF, hypertension and/or diabetes by the GP was calculated per subgroup of the test result. Differences were tested with a Chi-square test. All analyses were performed with SPSS 14.0.

## Results

### Characteristics of the respondents

**Figure 1:**
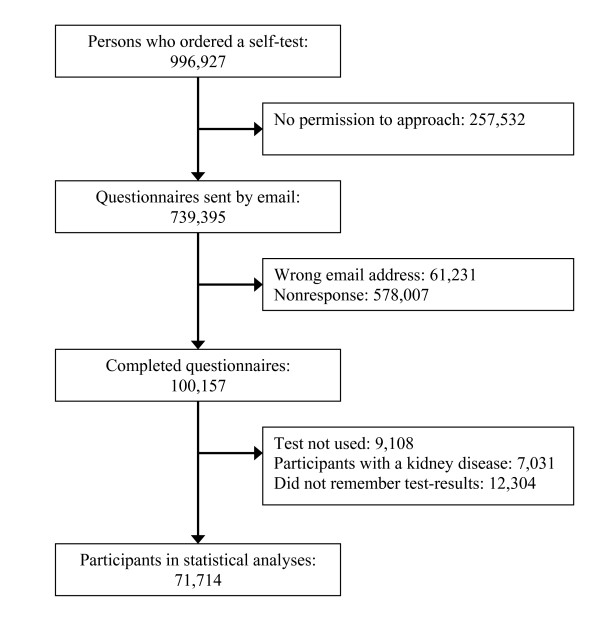
**Flow chart of participants**.

The results are based on the tests ordered during the first 30 days of the screening program. In this period, 996,927 self-tests were ordered via the Internet (7.8% of the adult population in the Netherlands). Persons who did not gave permission to be approached (n = 257,532), participants who did not complete the questionnaire (n = 639,238), participants with an existing kidney disease (n = 7,031), persons who did not use the test (n = 9,108) and persons who did not remember the results of the three dipstick tests (n = 12,304) were excluded from the analysis (see figure 1). The characteristics of the remaining respondents (n = 71,714), the total group of testers (n = 996,927) and the Dutch adult population are shown in table 1.

**Table 1:** Population characteristics

	**Respondents** **(n = 71,714)**	**Total group of testers** **(n = 996,927)**	Dutch adult population in 2006*
***Age categories***			
18-29 years (%)	4.6	12.2	18.4
30-39 years (%)	10.5	17.9	19.1
40-49 years (%)	19.3	23.3	19.9
50-59 years (%)	32.3	24.7	17.8
60-69 years (%)	25.8	15.5	12.0
70 years and older (%)	7.5	6.4	12.8
***Gender (% female)***	53.9	53.0	51.0
***Education***			
No/Low	6.4	--	--
Medium	61.8	--	--
High	31.8	--	--
***Alcohol use***			
No	53.0	--	--
Low	27.8	--	--
Medium	14.5	--	--
High	4.7	--	--
***BMI (mean (SD))***	25.5 (3.8)	--	--
***Smoking***			
Current	13.7	--	--
Former	49.4	--	--
Never	36.9	--	--
***Cardiovascular diseases***			
Hypertension (%)	19.3	--	--
Hypercholesterolaemia (%)	14.1	--	--
Previous myocardial infarction (%)	0.9	--	--
***Diabetes Mellitus***	5.7	--	--

* Source: Statistics Netherlands

The mean age of the respondents was 53 years and 54% was female. Compared with the total group of testers, the respondents were older and more often female. The higher mean age of the respondents was due to overrepresentation of persons of 50-59 years and 60-69 years. Persons who ordered a self-test were older compared with the Dutch adult population. Relatively more tests were ordered by persons of 40-49 years, 50-59 years and 60-69 years old. The mean BMI of the respondents was 25.5 kg/m^2^, 32% was highly educated, 19% used medium to high amounts of alcohol and 14% of the respondents was a current smoker.

### Self-reported results on the albuminuria self-test

Of the respondents, 56,885 (79.3%) reported a negative test result, 14,065 respondents reported a low-positive (19.6%) and 764 a high-positive test result (1.1%). In table 2 the percentage of participants with a positive test result are shown by age group and for persons with or without risk factors (diabetes mellitus and/or hypertension) at baseline. The percentage of respondents with a positive result decreased with increasing age. The highest proportion of high-positive testers was found in the age group 18-29 years old (3%; p < 0.001 compared with the other age groups) and the percentage of low-positive testers was highest in the age group 30-39 years old (32%; p < 0.001 compared with the age groups 40-49, 50-59, 60-69 and 70+ years old). The proportion of high-positive testers was highest in persons with diabetes mellitus (1.3%), but the proportion differed not statistically significant with persons with hypertension and persons without risk factors.

**Table 2:** Number of respondents with albuminuria by age and for persons with and without risk factors (percentages).

	**Albuminuria, % (95% CI)**		
	
	**Low-positive**	**High-positive**	**Positive total**
**Age groups (years)**			
			
18-29 (n = 3,303)	29.7 (28.1 - 31.3)	3.0 (2.4 - 3.5)	32.7 (31.1 - 34.3)
30-39 (n = 7,513)	32.4 (31.4 - 33.5)	1.9 (1.6 - 2.2)	34.3 (33.3 - 35.4)
40-49 (n = 13,847)	27.0 (26.2 - 27.7)	1.7 (1.5 - 1.9)	28.7 (27.9 - 29.4)
50-59 (n = 23,160)	17.2 (16.7 - 17.7)	0.7 (0.6 - 0.8)	17.9 (17.4 - 18.4)
60-69 (n = 18,519)	12.2 (11.7 - 12.6)	0.5 (0.4 - 0.6)	12.7 (12.2 - 13.2)
70+ (n = 5,372)	12.5 (11.6 - 13.3)	0.7 (0.5 - 1.0)	13.2 (12.3 - 14.1)

Total (n = 71,714)	19.6 (19.3 - 19.9)	1.1 (1.0 - 1.1)	20.7 (20.4 - 21.0)
			
**Presence of risk factors**			
			
No risk factors (n = 69,768)	19.6 (19.3 - 19.9)	1.1 (1.0 - 1.1)	20.7 (20.4 - 21.0)
Diabetes mellitus (n = 4,059)	18.9 (17.7 - 20.2)	1.3 (0.9 - 1.6)	20.2 (19.0 - 21.4)
Hypertension (n = 13,868)	18.1 (17.5 - 18.8)	1.1 (0.9 - 1.2)	19.2 (18.6 - 19.9)
Diabetes mellitus and hypertension (n = 1,946)	18.9 (17.2 - 20.7)	1.2 (0.7 - 1.7)	20.1 (18.3 - 21.9)

### GP visits

The number of GP visits and the number of newly detected diseases due to the screening program are shown in table 3. Only participants with a positive test result were recommended to visit a GP. Nevertheless, 0.5% of the participants with a negative test result also visited the GP and another 0.5% of them was planning to visit the GP later. Of the positive testers, 25% visited a GP after testing for albuminuria (25% and 33% of low-positive and high-positive testers, respectively) and 31% of the positive testers were planning to visit a GP at a later moment (31% and 27% of low-positive and high-positive testers, respectively).

**Table 3:** Number of reported newly detected diseases by a GP after the self-test program (n = 71,714)

	**Test result**			
		
	**Negative** **(n = 56,885)**	**Low-positive** **(n = 14,065)**	**High-positive** **(n = 764)**	**Total** **(n = 71,714)**
**GP visit?**				
Yes	288 (0.5%)	3,444 (24.5%)	251 (32.8%)	3,983 (5.6%)
**Newly detected disease?**				
No	280	3,285	225	3,790
Yes	8 (2.8%)	159 (4.6%)	26 (10.4%)	193 (4.8%)
**Which diseases?**				
Kidney disease	0	15	10	25
Hypertension	5	131	16	152
Diabetes Mellitus	3	25	3	31

In the group of 3,983 persons who visited a GP, 193 persons with a new disease were detected: 25 newly detected patients with a kidney disease, 152 newly detected hypertension patients and 31 newly detected diabetes mellitus patients. The percentage newly detected diseases was higher among the persons with a high-positive test result (10.4%) compared with the persons with a low-positive (4.6%) and a negative test result (2.8%) (p < 0.001).

## Discussion

During the first 30 days of the albuminuria screening program, 996,927 self-tests were ordered via the Internet. Of the testers who completed the questionnaire, 79% reported a negative test result, 20% reported a low-positive test result and 1% of the participants reported a high-positive test result. Of the positive testers, 25% visited a GP after testing for albuminuria and 31% of the positive testers were planning to visit a GP at a later moment. In the group of 3,983 persons who consulted a GP after applying the self-test, 193 persons with a newly detected disease were found: 25 cases of a kidney disease, 152 cases of hypertension and 31 cases of diabetes mellitus.

With the dipstick test used in this study it was possible to discriminate between four albumin concentrations (0, 30, 100 or 500 mg/l), which makes it difficult to distinguish between micro- and macroalbuminuria. Usually, microalbuminuria is defined as an urine albumin concentration between 20-200 mg/l and macroalbuminuria is defined as a concentration above 200 mg/l [11]. This suggests that the group high-positive testers (> 100 mg/l) mainly includes persons with macroalbuminuria. This is supported by the fact that the percentage of high-positive testers in this study is comparable with the prevalence of macroalbuminuria in population-based studies. For example, Garg et al found a macroalbuminurie prevalence of 1.3% in the U.S. population [12] and the PREVEND study in the Netherlands found a prevalence of 0.6% in a population-based cohort [13]. However, besides the high-positive testers, we also found an extremely high percentage respondents with a low-positive test result. It is likely that a part of the 20% low-positive test results are false positive. This may be caused by applying the test or reading the test results under the wrong conditions. Many people may have read the dipstick result in artificial light instead of day light, leading to misinterpretations. This hypothesis was tested by investigating the relation between the number of low-positive test results and darkness in the early morning hours. The percentage of low-positive testers appeared to be higher towards winter, as the days shortened (results not shown).

A positive result of the program is the high number of self-tests that were ordered. With one million tests ordered, we can assume that the program increased awareness among the Dutch population about the risk of renal diseases. Furthermore, the program resulted not only in a number of newly detected patients with a kidney disease, but also patients with chronic conditions that increase the risk of CRF were detected (diabetes mellitus and hypertension). Also some negative aspects can be discerned. When a part of the low-positive test results is false positive, this causes worries among the test users, unnecessary use of health care and therefore unnecessary costs. Moreover, the a-priori risk on CRF in the general population is low (similar to the prevalence in the general population of about 1%). In combination with the diagnostic value of the used dipstick tests, this will lead to only a small posterior risk on the disease after a positive test result. Under these circumstances, it is better to test in populations at high risk, such as patients with hypertension or diabetes mellitus. Finally, 75% of the positive testers had not consulted their general practitioner in relation to this test result in the study period, but some may have done that at a later stage. The reasons for this apparent lack of adequate reactions to unfavorable test results are unclear and call for further investigation.

It is obvious that we found the lowest percentages of people with a high-positive test result in the older age groups, in spite of an increased prevalence of cardiovascular risk factors and diabetes mellitus by age (data not shown), which is not in line with age-specific macroalbuminuria prevalence reported elsewhere [12]. This suggests that the albuminuria self-test has been ordered by relatively unhealthy young individuals and healthy older individuals. Older individuals with chronic diseases may already have regular checks with the GP. Furthermore, older people might be less familiar with Internet and/or have less access to Internet. Therefore, using a self-testing via the Internet for the detection of (chronic) disease could be a good method for detecting young people at risk.

This study has some limitations. First, the results are based on self-reported data from the participants of the screening program. It was not possible to validate the self-reported diagnoses of kidney diseases, cardiovascular diseases and diabetes mellitus and the results of the self-test. Second, only 13.5% of the participants responded on the questionnaire. It is unknown if the respondents are representative for the total group of participants of the CRF program. The motivation to complete the questionnaire may have been larger in testers with positive test results. On the other hand, the percentage high-positive testers was comparable with the prevalence of macroalbuminuria in other studies in the general population [12,13], which makes the occurrence of a selective response less likely.

The efficiency of such a campaign could be increased by embedding the testing in health care, e.g. the GP practice, to reduce the number of false-positive results and to ensure follow-up and treatment in case of a positive test result. The number of false-positive test results can be reduced 1) by testing in a population with a higher a priori risk on albuminuria, such as patients with hypertension and/or diabetes mellitus, and 2) when testing is performed by a health professional. The low number of GP consultations reduce the efficiency of the program, because positive testers who do not visit a GP, do not receive the treatment needed to prevent future kidney disease.

## Conclusion

It can be concluded that using a free self-test for screening of albuminuria had a large impact in the general population and resulted in a limited number of newly detected diseases. However there was a large number of probably false-positive test results and only a small part of the positive testers visited a GP for additional examination and/or treatment. Therefore, the efficiency of such a campaign could be increased by embedding the testing in health care, e.g. the GP practice, to reduce the number of false-positive results and to ensure follow-up and treatment in case of a positive test result.

## Competing interests

The authors declare that they have no competing interests.

## Authors' contributions

MMJN performed the study according to protocol, analyzed the data and wrote the manuscript. FGS and RAV initiated and supervised the study and developed the study protocol. All authors read and approved the final manuscript.

## Pre-publication history

The pre-publication history for this paper can be accessed here:


